# Revised mechanism of d-alanine incorporation into cell wall polymers in Gram-positive bacteria

**DOI:** 10.1099/mic.0.069898-0

**Published:** 2013-09

**Authors:** Nathalie T. Reichmann, Carolina Picarra Cassona, Angelika Gründling

**Affiliations:** Section of Microbiology and MRC Centre for Molecular Bacteriology and Infection, Imperial College London, London SW7 2AZ, UK

## Abstract

Teichoic acids (TAs) are important for growth, biofilm formation, adhesion and virulence of Gram-positive bacterial pathogens. The chemical structures of the TAs vary between bacteria, though they typically consist of zwitterionic polymers that are anchored to either the peptidoglycan layer as in the case of wall teichoic acid (WTA) or the cell membrane and named lipoteichoic acid (LTA). The polymers are modified with d-alanines and a lack of this decoration leads to increased susceptibility to cationic antimicrobial peptides. Four proteins, DltA–D, are essential for the incorporation of d-alanines into cell wall polymers and it has been established that DltA transfers d-alanines in the cytoplasm of the cell onto the carrier protein DltC. However, two conflicting models have been proposed for the remainder of the mechanism. Using a cellular protein localization and membrane topology analysis, we show here that DltC does not traverse the membrane and that DltD is anchored to the outside of the cell. These data are in agreement with the originally proposed model for d-alanine incorporation through a process that has been proposed to proceed via a d-alanine undecaprenyl phosphate membrane intermediate. Furthermore, we found that WTA isolated from a *Staphylococcus aureus* strain lacking LTA contains only a small amount of d-alanine, indicating that LTA has a role, either direct or indirect, in the efficient d-alanine incorporation into WTA in living cells.

## Introduction

The bacterial cell wall is a complex and highly organized structure that allows bacteria to interact with and protects them against hostile insults encountered in the environment. In Gram-positive bacteria, multi-functional teichoic acids (TAs) are key components of the cell wall. Many Gram-positive bacteria contain two types of TAs; wall teichoic acid (WTA), which is covalently linked to the peptidoglycan layer and lipoteichoic acid (LTA), which is embedded in the membrane via a lipid anchor (Reichmann & Gründling, 2011; Xia *et al.*, 2010a). Bacteria display diverse defects in the absence of either polymer and their combined absence is lethal to the cell (Oku *et al.*, 2009; Schirner *et al.*, 2009).

In *Staphylococcus aureus*, LTA is composed of a polyglycerolphosphate backbone chain that is linked via a glycolipid anchor to the outside of the membrane (Reichmann & Gründling, 2011). The backbone chain is polymerized on the outside of the cell by the LTA synthase enzyme LtaS using the membrane lipid phosphatidylglycerol as its substrate (Gründling & Schneewind, 2007b; Karatsa-Dodgson *et al.*, 2010; Koch *et al.*, 1984; Lu *et al.*, 2009). WTA in *S. aureus*, on the other hand, is composed of a ribitolphosphate backbone chain that is connected through a linker unit to muramic acid residues of peptidoglycan (Brown *et al.*, 2008; Neuhaus & Baddiley, 2003). WTA is further decorated with α- or β-*O*-*N*-acetylglucosamine residues and the enzymes required for this modification have been recently identified as TarM and TarS (Brown *et al.*, 2012; Xia *et al.*, 2010b). In *S. aureus* and many other Gram-positive bacteria, both polymers are further decorated with d-alanine esters, which confer a positive charge on the negative polymer (Neuhaus & Baddiley, 2003). Pulse–chase experiments using [^14^C]-alanine indicated that d-alanines are first incorporated into LTA (Haas *et al.*, 1984). Based on the observation that a decrease in radioactivity in the LTA fraction is followed by an increase in radioactivity in the WTA fraction, it has been suggested that d-alanine-LTA serves as donor for d-alanine substitutions in WTA (Haas *et al.*, 1984).

d-alanine modification of TAs is known to play an important role in the regulation of autolytic activity and binding of Mg^2+^ ions within the cell wall (Fischer *et al.*, 1981; Koprivnjak *et al.*, 2006; Lambert *et al.*, 1975). The absence of d-alanine esters leads to an increase in the susceptibility of bacteria to nisin, defensins and other cationic antimicrobial peptides and more rapid killing by phagocytic cells and neutrophils (Collins *et al.*, 2002; Kristian *et al.*, 2005; Peschel *et al.*, 1999; Poyart *et al.*, 2003; Walter *et al.*, 2007). *S. aureus* strains lacking d-alanine modifications are unable to colonize polystyrene or glass, are impaired in biofilm formation and show reduced adherence to nasal epithelial cells, and similar effects are observed in other bacteria (Gross *et al.*, 2001; Walter *et al.*, 2007; Weidenmaier *et al.*, 2004).

**Fig. 1.**
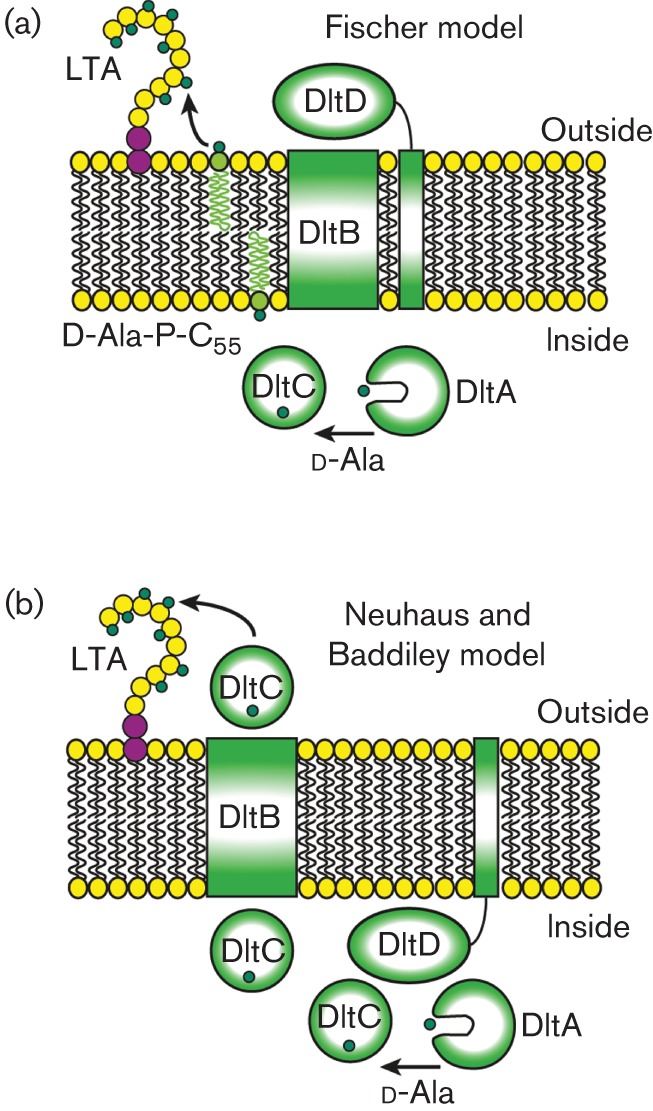
Models of d-alanine substitution of LTA. (a) Fischer model: DltA ligates d-alanine (small green circle) onto the carrier protein DltC. Next, it has been proposed that, with the aid of DltB, the d-alanine is transferred from DltC to undecaprenyl phosphate (C_55_-P) and traverses the membrane; however experimental evidence for such a membrane-linked intermediate is still lacking. DltD is then involved in the final transfer of the d-alanine to LTA on the outside of the cell. (b) Neuhaus and Baddiley model. For this model, it was suggested that DltD functions within the cell and increases the efficiency of the DltA-mediated ligation of d-alanine to DltC. The charged DltC protein then crosses the membrane with the help of DltB and transfers the d-alanine onto LTA.

Proteins required for the d-alanine incorporation into TAs are encoded in the *dlt* operon (Neuhaus & Baddiley, 2003; Neuhaus *et al.*, 1996) and in *S. aureus* this operon consists of five genes *dltXABCD* (Koprivnjak *et al.*, 2006). Based on the *dlt* operon in *Bacillus subtilis* only proteins encoded by *dltABCD* are thought to be essential for d-alanine incorporation (Koprivnjak *et al.*, 2006; Perego *et al.*, 1995). The function of DltA and DltC in this process has been established. DltA is a d-alanine-d-alanyl carrier protein ligase, which catalyses the adenylation of d-alanine and then the transfer of the activated amino acid onto the d-alanyl carrier protein DltC (Debabov *et al.*, 1996; Fischer, 1994; Heaton & Neuhaus, 1994). The roles played by DltB and DltD are less clear. DltB is a multi-membrane-spanning protein and hydropathy profiles indicate that DltD is also anchored to the membrane via an N-terminal hydrophobic sequence. Two models for the functions of these proteins have been proposed; Fischer and colleagues proposed that d-alanylation of TAs proceeds through a lipid-linked undecaprenyl phosphate (C_55_-P) intermediate. In this model, it was hypothesized that DltB facilitates the transfer of d-alanines from DltC to C_55_-P to produce d-Ala-P-C_55_ and possibly the subsequent transfer of this lipid-linked intermediate across the membrane (Fig. 1a) (Perego *et al.*, 1995). However, it should be noted that such a lipid-linked intermediate has not yet been confirmed experimentally. The final step in the d-alanylation process was proposed to be catalysed by DltD, which in this model functions on the outside of the cell (Perego *et al.*, 1995). The second model was formulated by Neuhaus and Baddiley following experiments performed by Debabov *et al.* on the DltD protein from *Lactobacillus rhamnosus* (Debabov *et al.*, 2000). Using purified proteins, it was shown that the rate of ligation of d-alanines from DltA to DltC increases twofold in the presence of DltD (Debabov *et al.*, 2000). This led to the hypothesis that DltD acts in the cytoplasm of the cell as a platform to bring DltA and DltC in close proximity allowing efficient charging of DltC with d-alanines (Fig. 1b). The charged DltC protein is then thought to translocate across the membrane via a channel formed by DltB and to transfer d-alanines in a final step onto LTA (Neuhaus & Baddiley, 2003).

In this study we revisited the mechanism of d-alanine incorporation into Gram-positive cell wall polymers. Using an *S. aureus* strain lacking LTA, we show that d-alanine is only very inefficiently incorporated into WTA, providing experimental evidence that in living cells d-alanine-LTA is required for the efficient incorporation of d-alanine into WTA. By performing a protein localization and membrane topology analysis in *S. aureus*, we show that DltC remains within the cell and that DltD is targeted to the outside of the cell, which is only consistent with the model proposed by Werner Fischer and colleagues. Based on our findings, we suggest that future studies addressing the mechanism of d-alanine incorporation into LTA should be designed based on the originally proposed model.

## Methods

### 

#### Bacterial stains and culture conditions.

Bacterial strains used in this study are listed in Table 1 and primers in Table 2. *Escherichia coli* strains were grown in LB medium and *S. aureus* strains in tryptic soy broth (TSB) or agar (TSA). All strains were grown at 37 °C and media were supplemented when appropriate with the antibiotics or inducers as listed in Table 1.

**Table 1.** Bacterial strains used in this study Antibiotics and inducers were used at the following concentrations: for *Escherichia* *coli* cultures, ampicillin (Amp^R^) 100 µg ml^−1^, kanamycin (Kan^R^) 30 µg ml^−1^; for *S.* *aureus* cultures, chloramphenicol (Cam^R^) 7.5 or 10 µg ml^−1^; anhydrotetracycline (Atet) at 200 ng ml^−1^.

Strain	Relevant features	Reference
***E. coli***		
XL1 Blue	Cloning strain; Tet^R^-ANG127	Stratagene
ANG284	p*itet* in XL1 Blue; pCL55 containing Atet inducible promoter; Amp^R^	Gründling & Schneewind (2007a)
ANG286	p*itet*-*lacZ* in XL1 Blue; Amp^R^	Lab. strain collection
ANG1103	pOK-*ltaS*-T300A in XL1 Blue; Kan^R^	Lu *et al.* (2009)
ANG1314	pUT18-*dltD* in XL1 Blue; Amp^R^	Lab. strain collection
ANG1482	p*itet*-*dltC*-His in XL1 Blue; *dltC-*His under Atet inducible promoter control; Amp^R^	This study
ANG1718	p*itet*-*dltD*-*lacZ* in XL1 Blue; *dltD* fused to *lacZ* under Atet inducible promoter control; Amp^R^	This study
ANG1719	p*itet*-*dltD_40 aa_*-*lacZ* in XL1 Blue; first 40 aa of *dltD* fused to *lacZ* under Atet inducible promoter control; Amp^R^	This study
ANG1720	p*itet*-3 aa-*lacZ* in XL1 Blue; first 3 aa of *dltD* fused to *lacZ* under Atet inducible promoter control; Amp^R^	This study
ANG1722	p*itet*-*aur_SS_*-*lacZ* in XL1 Blue; *aur* signal sequence fused to *lacZ* under Atet inducible promoter control; Amp^R^	This study
ANG1908	p*itet*-3 aa-*eltaS_T300A_*-His in XL1 Blue; first 3 aa of *dltD* fused to extracellular domain of *ltaS_T300A_* variant and His-tag under Atet inducible promoter control; Amp^R^	This study
ANG2021	p*itet*-*aur_SS_*-*eltaS_T300A_*-His in XL1 Blue; *aur* signal sequence fused to extracellular domain of *ltaS_T300A_* variant and His-tag under Atet inducible promoter control; Amp^R^	This study
ANG2022	p*itet*-*dltD_100 aa_*-*eltaS_T300A_*-His in XL1 Blue; first 100 aa of *dltD* fused to extracellular domain of *ltaS_T300A_* variant and His-tag under Atet inducible promoter control; Amp^R^	This study
ANG2041	p*itet*-*dltD_40 aa_*-*eltaS_T300A_*-His in XL1 Blue; first 40 aa of *dltD* fused to extracellular domain of *ltaS_T300A_* variant and His-tag under Atet inducible promoter; Amp^R^	This study
***S. aureus***		
SEJ1	RN4220Δ*spa*-ANG314	Gründling & Schneewind (2007a)
ANG1755	RN420Δ*spa*Δ*sbi*	Wörmann *et al.* (2011)
ANG1786	4S5; derivative of RN4220Δ*spa*Δ*ltaS* with mapped suppressor mutations, lacking LTA	Corrigan *et al.* (2011)
ANG1484	p*itet*-*dltC*-His integrated in strain ANG314; Cam^R^	This study
ANG1723	p*itet*-*dltD*-*lacZ* integrated in strain ANG314; Cam^R^	This study
ANG1724	p*itet*-*dltD_40 aa_*-*lacZ* integrated in strain ANG314; Cam^R^	This study
ANG1725	p*itet*-3 aa-*lacZ* integrated in strain ANG314; Cam^R^	This study
ANG1727	p*itet*-*aur_SS_*-*lacZ* integrated in strain ANG314; Cam^R^	This study
ANG1729	p*itet* integrated in strain ANG314; Cam^R^	This study
ANG2024	pCL55*itet*-3 aa-*eltaS_T300A_*-His integrated in strain ANG1755; Cam^R^	This study
ANG2025	pCL55*itet*-*aur_SS_*-*eltaS_T300A_*-His integrated in strain ANG1755; Cam^R^	This study
ANG2026	pCL55*itet*-*dltD_100 aa_*-*eltaS_T300A_*-His integrated in strain ANG1755; Cam^R^	This study
ANG2034	pCL55*itet* integrated in strain ANG1755; Cam^R^	This study
ANG2042	pCL55*itet*-*dltD_40 aa_*-*eltaS_T300A_*-His integrated in strain ANG1755; Cam^R^	This study

**Table 2.** Primers used in this study

Number	Primer	Sequence*
ANG420	3-*Bgl*II-His6-719	GAAGATCTTTAGTGATGGTGATGGTGATGACCTTTTTTAGAGTTTGCTTTAGGTCCTG
ANG721	5′-*Avr*II-DltC	CCGCCTAGGCTTATATAATAAAGGAGAATTTAATTATG
ANG722	3′-*Bgl*II-His-tag-DltC	GAAGATCTTTAGTGATGGTGATGGTGATGACCTCGTAACTCTTCTAATGCTTCAACG
ANG882	5′-*Avr*II-RBS-dltD for	CCGCCTAGGAAAAAATAAGGAGGAAAAAAAATGAAATTAAAACCTTTTTTACCC
ANG883	3′-*Sal*I-dltD rev	ACGCGTCGACATTTTTAGGTTTATCTACTTCAGG
ANG884	3′-*Sal*I-dltD 40 aa rev	ACGCGTCGACAGTTCTATTATCTTCTACAGTCTTTTC
ANG887	*Avr*II-RBS-3 aa-*Sal*I for	P-CTAGGAAAAAATAAGGAGGAAAAAAAATGAAATTAAAAG
ANG888	*Avr*II-RBS-3 aa-*Sal*I rev	P-TCGACTTTTAATTTCATTTTTTTTCCTCCTTATTTTTTC
ANG889	dltD 40 aa-eLtaS rev	GTCTGTCAGTTTCAGTTCTATTATCTTCTACAGTCTTTTC
ANG890	dltD 40 aa-eLtaS for	GATAATAGAACTGAAACTGACAGACCAGAATTATTAACACG
ANG891	dltD 100 aa-eLtaS rev	GTCTGTCAGTTTCAGAACCACCAGCACCTAATAAGAATGC
ANG892	dltD 100 aa-eLtaS for	GCTGGTGGTTCTGAAACTGACAGACCAGAATTATTAACACG
ANG1096	5′-*Avr*II-RBS-aur for	CCGCCTAGGAAAAAATAAGGAGGAAAAAAAATGAGGAAATTTTCAAGATATGC
ANG1097	3′-*Sal*I-aur rev	ACGCGTCGACCGCTAATGCTGCTGGTGATAAAG
ANG1138	*Avr*II-RBS-3 aa-eltaS	CCGCCTAGGAAAAAATAAGGAGGAAAAAAAATGAAATTAAAAGAAACTGACAGACCAGAATTATTAAC
ANG1216	aur sig seq-eltaS rev	GTCTGTCAGTTTCCGCTAATGCTGCTGGTGATAAAG
ANG1217	aur sig seq-eltaS for	GCAGCATTAGCGGAAACTGACAGACCAGAATTATTAAC

* Restriction sites in primer sequences are underlined.

#### Plasmid and strain construction.

Plasmid p*itet*-*dltC*-His was constructed for detection of DltC by Western blot analysis. The *dltC* gene from *S. aureus* Newman chromosomal DNA was amplified with primers 721/722, resulting in the addition of a C-terminal His-tag. The PCR product was digested with *Avr*II/*Bgl*II and ligated with plasmid p*itet*, which had been digested with the same enzymes. Plasmid p*itet*-*dltC*-His was initially obtained in *E. coli* XL1 Blue resulting in strain ANG1482 and subsequently integrated into the lipase gene *geh* of *S. aureus* RN4220Δ*spa* giving rise to strain ANG1484. For use as an empty vector control strain, p*itet* was introduced into *S. aureus* RN4220Δ*spa* resulting in strain ANG1729.

Plasmids p*itet*-*dltD*-*lacZ*, p*itet*-*dltD_40 aa_*-*lacZ*, p*itet*-3 aa-*lacZ*, and p*itet*-*aur_SS_*-*lacZ* were constructed for membrane topology studies in *S. aureus*. Plasmids p*itet*-*dltD*-*lacZ* and p*itet*-*dltD_40 aa_*-*lacZ* were constructed by amplifying the appropriate *dltD* sequence from plasmid pUT18-*dltD* (ANG1314) with primers 882/883 and 882/884, respectively. Following digestion with *Avr*II/*Sal*I, PCR products were ligated with p*itet*-*lacZ* (ANG286), which had been digested with the same enzymes. Plasmids p*itet*-*dltD*-*lacZ* and p*itet*-*dltD_40 aa_*-*lacZ* were initially obtained in *E. coli* XL1 Blue yielding strains ANG1718 and ANG1719, and subsequently transformed into *S. aureus* RN4220Δ*spa* resulting in strains ANG1723 and ANG1724, respectively. To construct plasmid p*itet*-3 aa-*lacZ*, the sequence encoding the ribosome-binding site and the first three amino acids of DltD was generated by annealing the primers 887/888. The annealed primers were ligated with p*itet*-*lacZ*, which had been digested with *Avr*II/*Sal*I. Plasmid p*itet*-3 aa-*lacZ* was initially obtained in *E. coli* XL1 Blue giving rise to strain ANG1720 and subsequently transformed into *S. aureus* RN4220Δ*spa* yielding strain ANG1725. Plasmid p*itet*-*aur_SS_*-*lacZ* was constructed by amplifying the sequence encoding the signal sequence of aureolysin (*aur_SS_*) from *S. aureus* Newman chromosomal DNA with primers 1096/1097. The PCR product was digested with *Avr*II/*Sal*I and ligated with plasmid p*itet*-*lacZ*, which had been digested with the same enzymes. Plasmid p*itet*-*aur_SS_*-*lacZ* was initially obtained in *E. coli* XL1 Blue resulting in strain ANG1722 and subsequently transformed into *S. aureus* RN4220Δ*spa* giving rise to strain ANG1727.

Fusions to the extracellular domain of the inactive LtaS variant eLtaS_T300A_ with a C-terminal 6×His-tag were used for membrane topology studies. Plasmids p*itet*-*dltD_40 aa_*-*eltaS_T300A_*-His, p*itet*-*dltD_100 aa_*-*eltaS_T300A_*-His, p*itet*-3 aa-*eltaS_T300A_*-His and p*itet*-*aur_SS_*-*eltaS_T300A_*-His were generated for this purpose. Plasmids p*itet*-*dltD_40 aa_*-*eltaS_T300A_*-His and p*itet*-*dltD_100 aa_*-*eltaS_T300A_*-His were constructed by amplifying the sequence encoding the first 40 or 100 amino acids of DltD from pUT18-*dltD* (ANG1314) with primers 882/889 and 882/891 and the *eltaS_T300A_*-His sequence from pOK-*ltaS*-T300A (ANG1103) using primers 890/420 and 892/420, respectively. The resulting products were fused by splicing by overlap extension (SOE) PCR using primers 882/420. The final PCR products were digested with *Avr*II/*Bgl*II and ligated with plasmid p*itet*, which had been digested with the same enzymes. Plasmids p*itet*-*dltD_40 aa_*-*eltaS_T300A_*-His and p*itet*-*dltD_100 aa_*-*eltaS_T300A_*-His were initially transformed into *E. coli* XL1 Blue resulting in strains ANG2041 and ANG2022, and subsequently transformed into *S. aureus* RN4220Δ*spa*Δ*sbi* yielding strains ANG2042 and ANG2026, respectively. In order to generate plasmid p*itet*-3 aa-*eltaS_T300A_*-His, the *eltaS_T300A_* sequence was amplified from pOK-*ltaS*-T300A (ANG1103) with primers 1138/420, resulting in the addition of the ribosome-binding site and sequences encoding the first three amino acids of DltD to the 5′ end and a 6×His-tag to the 3′ end. This PCR product was digested with *Avr*II/*Bgl*II and ligated with plasmid p*itet*, which had been digested with the same enzymes. Plasmid p*itet*-3 aa-*eltaS_T300A_*-His was initially transformed into *E. coli* XL1 Blue giving rise to strain ANG1908 and subsequently transformed into *S. aureus* RN4220Δ*spa*Δ*sbi* yielding strain ANG2024. Plasmid p*itet*-*aur_SS_*-*eltaS_T300A_*-His was constructed by amplifying the sequence encoding the signal sequence of aureolysin (*aur_SS_*) from p*itet*-*aur_SS_*-*lacZ* (ANG1722) with primers 1096/1216 and the *eltaS_T300A_*-His sequence from pOK-*ltaS*-T300A (ANG1103) with primers 1217/420. The resulting products were fused by SOE PCR using primers 1096/420. The final PCR product was digested with *Avr*II/*Bgl*II and ligated with plasmid p*itet*, which had been digested with the same enzymes. Plasmid p*itet*-*aur_SS_*-*eltaS_T300A_*-His was initially transformed into *E. coli* XL1 Blue resulting in strain ANG2021 and subsequently transformed into *S. aureus* RN4220Δ*spa*Δ*sbi* yielding strain ANG2025. The sequences of all inserts were verified by fluorescent automatic sequencing at the MRC Clinical Sciences Centre at Imperial College London.

#### Cell fractionation and Western blot analysis.

For DltC-His detection, overnight cultures of *S. aureus* were diluted 1 : 100 into 5 ml TSB medium with anhydrotetracycline (Atet) and grown for 4.5 h at 37 °C with shaking. For cell fractionation into cytoplasm plus membrane (cell), cell wall and supernatant, cells of a 1 ml culture were pelleted by centrifugation at 7000 ***g*** for 15 min. Nine hundred microlitre of the supernatant was precipitated with trichloroacetic acid (TCA) as previously described (Wörmann *et al.*, 2011). The remaining supernatant was removed from the cell pellet and bacteria suspended in 1 ml osmotically stabilizing lysis buffer (50 mM Tris/HCl pH 7.5, 20 mM MgCl_2_, 30 % raffinose and 200 µg ml^−1^ lysostaphin) and incubated at 37 °C for 30 min. The protoplasts were collected by centrifugation at 6000 ***g*** for 20 min and suspended in protein sample buffer, yielding the cell fraction (cytoplasm and membrane). Nine hundred microlitre of the supernatant (cell wall fraction) was TCA precipitated as described above. For detection of the eLtaS_T300A_-His fusion proteins, the supernatant fraction was prepared as described above and for the cell fraction the bacterial pellet from 1 ml culture was suspended in 1 ml lysis buffer (100 mM Tris/HCl pH 7.5, 10 mM MgCl_2_, 200 µg ml^−1^ lysostaphin) and incubated for 30 min at 37 °C. The sample was subsequently centrifuged at 17000 g for 5 min and 900 µl of the supernatant TCA precipitated. All samples were suspended in protein sample buffer normalized for culture OD_600_ readings; that is a culture with an OD_600_ of 3 was suspended in 45 µl sample buffer. Samples were boiled for 15 min and centrifuged at 17 000 ***g*** for 5 min, and 10 µl aliquots were separated on 15 % or 10% SDS-PAGE gels for Western blot analysis. His-tagged proteins were detected with HRP-conjugated anti-His antibody (Sigma) at a 1 : 10 000 dilution. The control proteins L6 (cytoplasmic) (gift from O. Schneewind, University of Chicago, USA), StrA (membrane) (Mazmanian *et al.*, 2000), SdrD (cell wall) (DeDent *et al.*, 2008) and α-haemolysin (Hla) (supernatant) (Bubeck Wardenburg & Schneewind, 2008) were detected with respective primary antibodies used at a 1 : 20 000 dilution and an HRP-conjugated anti-rabbit IgG secondary antibody at a 1 : 10 000 dilution. All experiments were performed at least three times and representative Western blots are shown.

#### β-Galactosidase activity assay.

Overnight cultures of *S. aureus* were diluted 1 : 100 in 5 ml TSB containing 200 ng ml^−1^ anhydrotetracycline (Atet) and grown for 4 h at 37 °C with shaking. Cells from a 1 ml culture aliquot were collected by centrifugation at 17 000 ***g*** for 10 min and the pellet was frozen at −20 °C overnight. Samples were thawed and pellets suspended in 1 ml ABT buffer (60 mM K_2_HPO_4_, 40 mM KHPO_4_, 100 mM NaCl, pH 7, 1 % Triton X-100) containing 20 µg ml^−1^ lysostaphin and incubated at 37 °C for 30 min. Cell debris were pelleted by centrifugation at 17 000 ***g*** for 10 min and 100 µl of the supernatant was added to 20 µl 0.4 mg ml^−1^ 4-methylumbelliferyl β-d-galactopyranoside (MUG) in a black 96-well plate. As background control 100 µl ABT was added to 20 µl MUG solution. Following incubation in the dark at room temperature for 1 h, a 20 µl sample was mixed with 180 µl ABT buffer and fluorescence readings were detected at an excitation wavelength of 336 nm and emission wavelength of 445 nm. A standard curve was generated using serial dilutions of 4-methylumbelliferone in ABT buffer at known concentration. Subsequently these values were used to determine the concentration (μM) of product in each sample and results are given as μM per OD_600_ of 1. The experiment was performed in triplicate and mean values and standard deviations plotted.

#### WTA purification and NMR analysis.

Purification of peptidoglycan-WTA complexes was performed as previously described (Bernal *et al.*, 2009; Kopp *et al.*, 1996; Strandén *et al.*, 1997) with slight modifications. Overnight cultures of *S. aureus* strain RN4220Δ*spa* or the LTA negative strain 4S5 were diluted into 5 l TSB to obtain an initial OD_600_ of 0.06 and grown at 37 °C with shaking until an OD_600_ of approximately 3. Bacteria were collected by centrifugation at 6000 ***g*** for 10 min and washed once with 1 M NaCl. Cells were suspended in 40 ml 1 M NaCl, disrupted with 0.1 mm glass beads using a bead beater and the broken cells collected by centrifugation for 40 min at 13 300 ***g***. The cell wall material was washed once with 1 M NaCl, three times with 0.5 % SDS and three times with water. The material was suspended in 30 ml water and incubated for 30 min at 60 °C with gentle stirring. The cell wall material was recovered by centrifugation at 34 600 ***g*** for 20 min, washed once with water and recovered again by centrifugation. The final pellet was suspended in 20 ml 0.15 mM Tris/HCl pH 7.0 containing 0.2 mg ml^−1^ trypsin and incubated at 37 °C for 18 h. The material was recovered by centrifugation and washed with 1 M Tris/HCl pH 7.0, 1 M Tris/HCl pH 7.0 containing 1 M NaCl, 1 M Tris/HCl pH 7.0 and three times with water. To hydrolyse WTA from peptidoglycan, the cell wall material was incubated in 10 % TCA at 4 °C for 18 h. The peptidoglycan was removed by centrifugation at 9700 ***g*** for 45 min. WTA was precipitated with 0.1 volume of 3 M sodium acetate pH 5.2 and 3 volumes of 95 % ice-cold ethanol and held overnight at −80 °C. The following day, the WTA was precipitated by centrifugation at 9700 ***g*** for 30 min and was washed five times with 95 % ethanol. Following the last wash step the pellet was air-dried and the WTA was suspended in 500 µl of water and lyophilized overnight. For the NMR analysis, 6 mg of WTA was suspended and lyophilized twice in 99.96 % D_2_O and finally suspended in 99.99 % D_2_O. Spectra were acquired on a 600 MHz Bruker AVANCE III spectrometer equipped with TCI cryoprobe and processed using Bruker TopSpin 3.1 software. The experiment was performed in triplicate.

## Results

### DltC remains within the cell

**Fig. 2.**
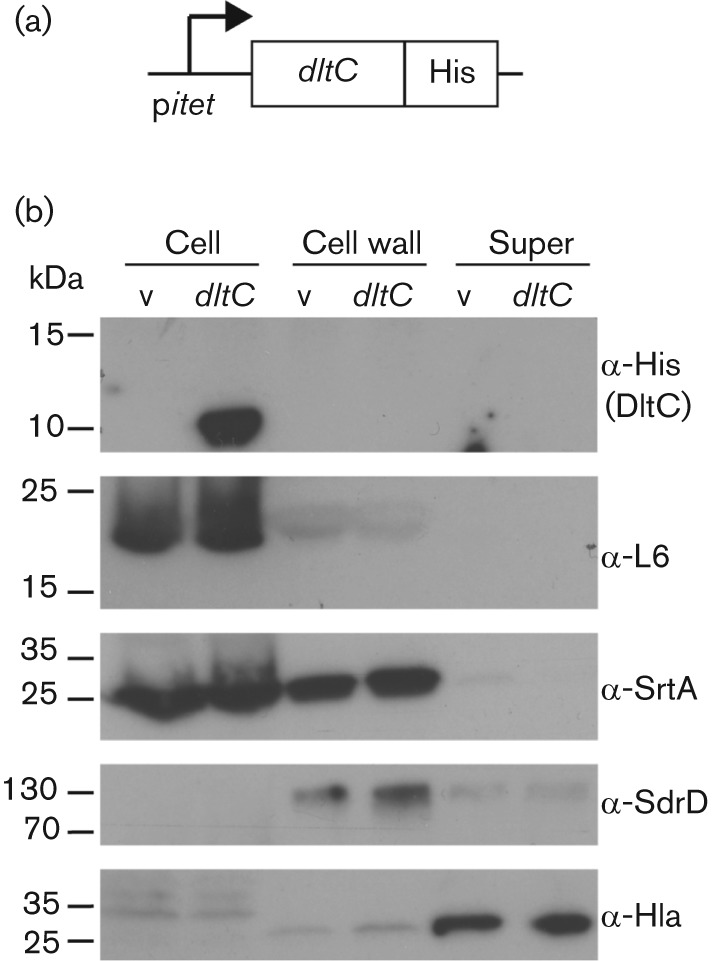
Cellular localization of DltC as assessed by Western blot. (a) Schematic representation of *dltC* expression construct. The gene encoding a C-terminally His-tagged version of DltC is placed under the inducible tetracycline promoter (p*itet*) and its expression induced by the addition of the less toxic tetracycline-derivative Atet. (b) Determination of DltC localization by Western blot analysis. *S. aureus* strain ANG1729 containing the empty vector p*itet* (v) or strain ANG1484 containing p*itet*-*dltC*-His (*dltC)* were grown for 4.5 h at 37 °C in the presence of Atet and samples were subsequently separated into cell (cytoplasm and membrane), cell wall and supernatant (super) fractions and analysed by Western blot using a His-tag specific antibody for the detection of DltC or antibodies specific for the ribosomal protein L6 (cytoplasmic), the sortase enzyme SrtA (membrane), the cell wall anchored protein SdrD (cell wall) and the secreted α-haemolysin Hla (supernatant). The molecular mass of protein standards is indicated on the left of each panel. The experiment was performed in triplicate and a representative blot is shown.

A key difference between the proposed models for d-alanine incorporation into TAs is the cellular location of the small carrier protein DltC. In the Fischer model this protein remains within the cytoplasm of the cell, while in the Neuhaus and Baddiley model the protein crosses the membrane and is at least transiently located on the outside of the cell (Fig. 1). To distinguish between these possibilities, we set out to determine the cellular location of DltC. To this end, *S. aureus* strain ANG1484 was constructed for expression of DltC with a C-terminal His-tag from the anhydrotetracycline (Atet) inducible *itet* promoter (Fig. 2a). As a control, strain ANG1729 containing an empty vector was used. Both strains were grown to mid-exponential phase in the presence of Atet and subsequently cell (cytoplasm and membrane), cell wall and supernatant fractions prepared. The DltC protein was detected by Western blot using a His-tag specific antibody and detection of the cytoplasmically located ribosomal protein L6, the membrane protein SrtA, the cell wall protein SdrD and the secreted α-haemolysin (α-Hla) provided fractionation controls (Fig. 2b). DltC was only detected in the cell fraction and no signal was observed in the cell wall or supernatant fraction (Fig. 2b). These data are in better agreement with the model proposed by Werner Fischer and colleagues.

### DltD is oriented towards the outside of the cell

**Fig. 3.**
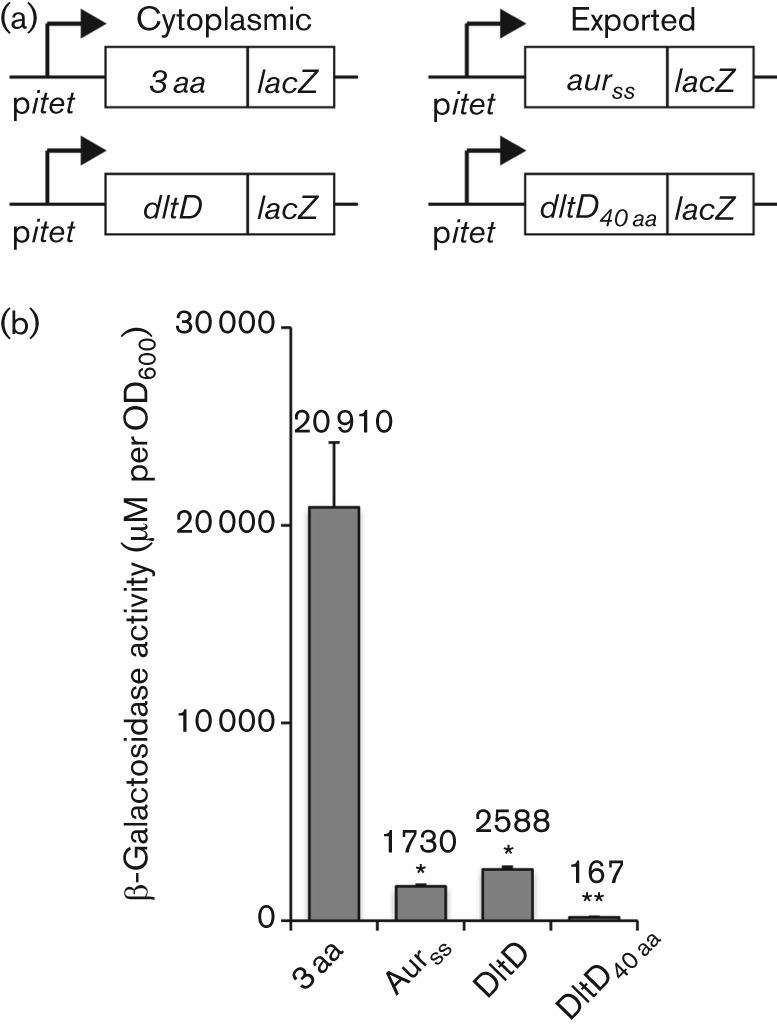
Membrane topology of DltD as assessed by LacZ fusions and β-galactosidase activity assays. (a) Schematic representation of the different *lacZ* fusions. The *dltD* gene or the sequence encoding the first 40 aa of *dltD* was cloned into p*itet*-*lacZ* giving rise to *dltD-lacZ* fusions expressed from the Atet inducible promoter. Sequences encoding the first three amino acids of *dltD* (3 aa) and the signal sequence of aureolysin (*aur_SS_*) were cloned upstream of *lacZ*, providing cytoplasmic and secreted controls, respectively. (b) Determination of β-galactosidase activity. *S. aureus* strains containing p*itet*-3 aa*-lacZ*, p*itet*-*aur_SS_-lacZ*, p*itet*-*dltD*-*lacZ* (*dltD)* and p*itet*-*dltD_40 aa_*-*lacZ* were grown for 4 h at 37 °C in the presence of 200 ng ml^−1^ Atet and samples were prepared for β-galactosidase activity assays as described in the Methods section. The assay was performed in triplicate and the mean values and standard deviations were plotted. Activity is given as μM per OD_600_ unit. *t*-Test analysis was performed and values which are significantly different from the positive control (3 aa) are indicated with asterisks as follows: *P<0.05, **P<0.01.

DltD contains an N-terminal hydrophobic domain, which serves to anchor the protein in the membrane. According to the Neuhaus and Baddiley model, the protein is anchored in the membrane with an N terminus out/C terminus in topology, while in the Fischer model DltD has the opposite orientation (Fig. 1). To investigate the membrane topology of DltD, LacZ fusions were constructed with the first 40 amino acids or full-length DltD and the fusion proteins were expressed in *S. aureus* from the inducible *itet* promoter by the addition of Atet (Fig. 3a). LacZ is only active within the cytoplasm of the cell and LacZ fusions with the first three amino acids of DltD or the signal sequence of aureolysin served as cytoplasmic or secreted controls, yielding as expected high or low β-galactosidase activities, respectively (Fig. 3b). Expression of the 40 amino acid or full-length DltD–LacZ fusion proteins yielded very low activity, indicating that DltD has an N terminus in/C terminus out membrane topology consistent with the Fischer model (Fig. 3b).

**Fig. 4.**
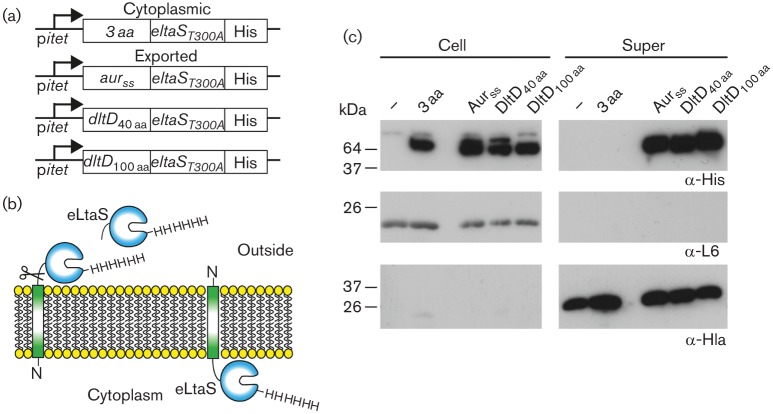
Membrane topology of DltD as assessed by Western blot analysis using eLtaS_T300A_ fusion proteins. (a) Schematic representation of the vectors for the expression of the eLtaS_T300A_-His fusions. Sequences encoding the first three amino acids of *dltD* (3 aa) (cytoplasmic control) or the signal sequence of aureolysin (*aur_SS_*) (secreted control) as well as the first 40 aa or first 100 aa of *dltD* were fused to *eltaS_T300A_-*His and expressed from the Atet inducible promoter in *S. aureus*. (b) Schematic representation of the eLtaS_T300A_-His fusion protein. A fusion protein with an N terminus in/C terminus out membrane topology (left) will lead to secretion of eLtaS_T300A_-His into the supernatant, while a fusion protein with an N out/C in membrane topology (right) will cause the retention of eLtaS_T300A_-His in the cytoplasm of the cell. (c) Detection of the eLtaS_T300A_-His protein fusions by Western blot. *S.* *aureus* strains containing the empty vector p*itet* (–), p*itet*-3 aa-*eltaS_T300A_-*His, p*itet*-*aur_SS_*-*eltaS_T300A_-*His, p*itet*-*dltD_40 aa_-eltaS_T300A_-*His or p*itet*-*dltD_100 aa_*-*eltaS_T300A_* were grown in the presence of 200 ng ml^−1^ Atet for 4 h at 37 °C and subsequently cell (Cell) and supernatant (Super) fraction samples were prepared and analysed by Western blot. The HRP-conjugated anti-His antibody was used at a 1 : 10 000 dilution. Rabbit polyclonal antibodies against the control proteins L6 (cytoplasmic) and Hla (supernatant) were used at a 1 : 20 000 dilution, followed by incubation with HRP-conjugated anti-rabbit IgG antibodies used at a 1 : 10 000 dilution. This experiment was performed in triplicate and a representative result is shown.

To verify this result, protein fusions were designed with the stably folded extracellular eLtaS domain of the LTA synthase enzyme LtaS. It has been previously shown that when this domain is fused to a signal peptide or transmembrane domain with an N terminus in/C terminus out topology, it is cleaved by the signal peptidase and can be readily detected in the culture supernatant by Western blot (Wörmann *et al.*, 2011). The enzymatically inactive eLtaS_T300A_ variant containing a C-terminal His-tag was fused to the first 40 or 100 amino acids of DltD and fusions with the first three amino acids of DltD or the aureolysin signal peptide were produced as cytoplasmic or exported controls, respectively (Fig. 4a, b). Cell and supernatant fractions were prepared from *S. aureus* strains containing an empty vector (–) or vectors for the expression of the different fusions proteins. The fusion proteins were detected by Western blot using an anti-His-tag antibody and the fractionation technique was verified using antibodies specific for the cytoplasmically located ribosomal protein L6 or the α-haemolysin (Fig. 4c). As expected, eLtaS_T300A_-His was detected in the supernatant fraction for the aureolysin signal peptide control fusion. Proteins were also detected in the supernatant fraction for the 40 and 100 amino acid DltD fusions, while no signals were detected for samples isolated from the empty vector containing control strain or a strain expressing the cytoplasmic 3 aa-eLtaS_T300A_-His control fusion, the latter of which was detected in the cell fraction (Fig. 4c). Double bands were also observed in the cell fraction for the aur_SS_-eLtaS_T300A_, DltD_40 aa_ and DltD_100 aa_ fusion proteins (Fig. 4c). The less intense upper bands, which differ in size between the different fusion proteins, likely correspond to the full-length proteins. The lower protein bands, which are of similar size for all fusion proteins, are likely fusion protein fragments that have been transported to the outside of the cell, cleaved by the signal peptidase, but not yet released from the cell wall. Taken together, the Western blot data and the LacZ activity assays indicate that DltD has an N terminus in/C terminus out membrane topology, consistent with the Fischer model and a function for DltD on the outside of the cell.

### LTA is required for efficient incorporation of d-alanine into WTA

**Fig. 5.**
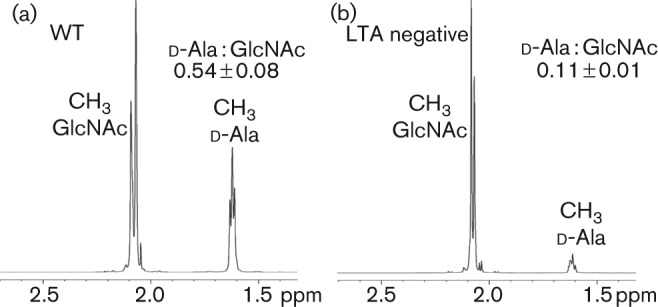
NMR analysis of WTA isolated from WT and LTA negative *S. aureus* strains. *S. aureus* strains SEJ1 (WT) and 4S5 (LTA negative) were grown to mid-exponential phase and WTA was purified as described in the Methods section. Six milligrams of dried WTA were suspended in 99.99 % D_2_O and 1-D proton spectra acquired at 600 MHz. The experiment was performed in triplicate and representative spectra are shown. The ratio of the d-Ala to GlcNAc signal is 0.54±0.08 for WTA isolated from a WT strain and 0.11±0.01 for WTA isolated from the LTA negative strain. A two-tailed unequal variance *t*-test gave a *P*-value <0.01 indicating statistically significant differences. Peaks are annotated as previously described (Bernal *et al.*, 2009), and the full spectra are shown in Fig. S1.

Disruption of *dltA-D* in *S. aureus* results in a lack of d-alanine substitutions not only in LTA, but also in WTA (Perego *et al.*, 1995). While this may imply that the Dlt proteins are involved in the transfer of d-alanine onto WTA, studies using [^14^C]-alanine have shown that when the radioactivity is lost from the LTA fraction it increases in the WTA fraction (Haas *et al.*, 1984; Koch *et al.*, 1985). Based on these findings, it has been proposed that d-alanine-LTA is the d-alanine donor for WTA. To address this further, d-alanine incorporation into WTA was investigated in the LTA negative (Δ*ltaS*) *S. aureus* strain 4S5. Of note, in a previous study a whole genome sequence analysis was performed on strain 4S5, which revealed that this strain contains an intact *dlt* operon (Corrigan *et al.*, 2011). To this end, cultures of the wild-type *S. aureus* control strain and the LTA deficient strain 4S5 were grown to mid-exponential phase, cell walls were purified and the WTA was released in acid conditions to retain the d-alanine modifications. The purified WTA material was subsequently analysed by 1-D proton NMR. Clear signals derived from protons from the d-alanine and *N*-acetylglucosamine (GlcNAc) modifications were observed in WTA samples isolated from the wild-type strain (Fig. 5 and Fig. S1, available in *Microbiology* Online). As previously reported (Bernal *et al.*, 2009), the ratio of the signal from the three methyl group protons of d-Ala at 1.6 p.p.m. to GlcNAc at 2.1 p.p.m. was 0.54±0.08 for WTA isolated from a WT strain (Fig. 5a and Fig. S1). On the other hand, the WTA isolated from the LTA negative strain showed a drastically and statistically significant reduction in the D-alanine specific signal, yielding a d-Ala to GlcNAc ratio of 0.11±0.01 (Fig. 5b and Fig S1). These results show that LTA is important for efficient d-alanylation of WTA and are consistent with a model in which d-alanine-LTA serves as major d-alanine donor for WTA. However, an alternative mechanism in which LTA has an indirect role in the d-alanylation of WTA could also take place, as discussed below.

## Discussion

In this study, we revisited the Dlt-protein-mediated d-alanine incorporation mechanism into TAs of Gram-positive bacteria. Using an *in vitro* assay system, it has been reported in a previous study that alanine can transfer without the requirement of ATP and in an enzyme- and Dlt-protein-independent manner between LTA molecules (Childs *et al.*, 1985). In a second study, it has been proposed that d-alanine-LTA is the d-alanine donor for WTA; however in this case it has been invoked that the process is enzyme-catalysed (Haas *et al.*, 1984). While in this study we did not address whether or not the redistribution of d-alanines between TAs is enzyme-catalysed, the recent description of an LTA negative *S. aureus* strain allowed us to investigate the requirement of LTA for the d-alanylation of WTA further. We show here that the d-alanine content in WTA is drastically reduced in the absence of LTA, which is in agreement with the hypothesis that d-alanylated LTA is important for efficient modification of WTA (Fig. 5). However, our data also showed that, even in the absence of LTA, some d-alanine is still present in WTA. This might suggest that WTA polymers, which have been transported to the outside of the membrane but are still linked to the undecaprenyl phosphate membrane carrier, can, although very inefficiently, serve as acceptor molecules for d-alanine modification by the Dlt system. This may in part reflect the transient location of lipid-carrier anchored WTA at the membrane–wall interface prior to incorporation into the cell wall. An LTA negative *S. aureus* strain has usually a severe growth defect (Gründling & Schneewind, 2007b); however the LTA negative *S. aureus* strain used in this study survives in the absence of LTA and grows nearly like a wild-type strain through the acquisition of compensatory mutations (Corrigan *et al.*, 2011). Genome sequence analysis confirmed that the *dlt* operon is intact (Corrigan *et al.*, 2011), which could have been an alternative explanation for the reduced levels of d-alanine in WTA in this strain. However, it cannot be completely ruled out that the observed reduction of d-alanine in WTA is not due to an incorrect assembly of the DltB or DltD proteins in the membrane caused by a lack of LTA rather than that, as we suggest, d-alanine LTA is the major donor of d-alanine for WTA.

Two conflicting models have been proposed for the incorporation of d-alanine into TAs although the initial steps are identical in both models. It has been well established that the cytoplasmic d-alanine d-alanyl carrier protein ligase DltA, which shows homology to the acetyl coenzyme A synthases, uses ATP to activate d-alanine to form d-alanyl-AMP. In a second step, DltA then transfers this intermediate onto the small d-alanyl carrier protein DltC, where the d-alanine is bound through a thiol ester bond to the phosphopantetheine prosthetic group in DltC (Du *et al.*, 2008; Neuhaus & Baddiley, 2003; Osman *et al.*, 2009; Yonus *et al.*, 2008). DltC shows sequence and structural homology to acyl carrier proteins (ACPs), which in bacteria function in the cytoplasm of the cell and are involved in fatty acid and polyketide biosynthesis pathways (Volkman *et al.*, 2001). In this study, we show that DltC does not cross the membrane (Fig. 2) and therefore it is unlikely that the protein is involved in the final d-alanylation step of LTA on the outside of the cell. Furthermore, the results presented in this study indicate that DltD has an N terminus in/C terminus out membrane topology (Fig. 3). This places the functional part of the protein on the outside of the cell and suggests that DltD aids in the final step of the d-alanine incorporation into LTA. Both of these findings are only consistent with the model proposed by Werner Fischer and colleagues (Fig. 1a). According to the Fischer model, once DltC is charged with a d-alanine, the multi-membrane-spanning protein DltB transfers d-alanine from DltC to C_55_-P, resulting in the formation of a d-alanine-P-C_55_ membrane intermediate. This hypothesis is based on the proposed model for the glycosylation process of LTA (Fischer, 1994), though this membrane intermediate has never been experimentally confirmed. Based on the hydropathy profile and the TMHMM membrane topology prediction program, DltB assembles as a ten transmembrane helix protein with both N- and C-termini located on the outside. DltB has been grouped among membrane-bound *O*-acyltransferases (MBOAT) family proteins, a group of enzymes that transfer organic acids onto hydroxyl groups of membrane-embedded components (Hofmann, 2000). Some of the best-characterized members of MBOAT proteins are enzymes involved in the reacylation of lysophospholipids (Shindou *et al.*, 2009). This would be consistent with the idea that DltB does not merely form a membrane channel but also contains enzymatic activity, which will be necessary for the formation of a membrane-linked d-alanine intermediate. In addition, it cannot be ruled out that DltB plays a role together with DltD in the final cleavage and ligation of d-alanine to LTA.

The *dlt* operon in *S. aureus* encodes a fifth protein, DltX, which is a small protein with an expected size of 5.9 kDa. DltX has been annotated to belong to the DUF3687 superfamily of proteins and currently 185 proteins with this domain are listed in Pfam. With two exceptions, these proteins are encoded immediately upstream of *dltA* in *S. aureus* strains, other *Staphylococcus* sp. and several other *Firmicutes* including some *Bacillus*, *Lactobacillus*, *Listeria*, *Streptococcus* and *Enterococcus* sp. However, additional work is needed to determine the function of DltX and establish whether or not this protein is involved in the d-alanylation process in *S. aureus* or other *Firmicutes*.

Recently it has been shown that the lipopolysaccharide (LPS) in the Gram-negative bacterial pathogen *Vibrio cholera* O1 El Tor is also modified with amino acids, specifically glycine or diglycine residues (Hankins *et al.*, 2012). The machinery used shows similarities to the d-alanine modification system of TAs in Gram-positive bacteria. AlmF, which shows homology to DltA, activates the glycine residues using ATP and ligates it to AlmE. AlmE does not show homology on the sequence level with DltC, but shows functional and likely structural homology to DltC. Once AlmE is charged with a glycine residue, it is transferred by AlmG, which contains a lysophospholipid acyltransferase (LPLAT) domain, onto LPS. Again, AlmG does not share any sequence homology with DltB, but both proteins are predicted to belong to acyltransferase enzyme families, and therefore it seems likely that as functional homologues these proteins are required for the transfer of the amino acids from the charged carrier protein to lipid-linked acceptor molecules (Hankins *et al.*, 2012).

While additional work is needed to fully elucidate the mechanism of d-alanine incorporation into Gram-positive cell wall polymers, our cellular location and membrane topology studies on the *S. aureus* DltC and DltD proteins are in better agreement with the model proposed by Werner Fischer and colleagues. Therefore, we suggest that future investigations into the d-alanine incorporation mechanism should be designed with this model in mind. Our preliminary findings indicate that proteins involved in the LTA synthesis and the d-alanylation process may physically interact within bacterial cells and it will be interesting to investigate in future studies the spatial and temporal coordination of the cell wall polymer synthesis machineries and proteins responsible for their modification.
